# Filled skutterudite structure of europium ruthenium polyphosphide, EuRu_4_P_12_

**DOI:** 10.1107/S1600536810000589

**Published:** 2010-01-13

**Authors:** Isao Kagomiya, Shiro Funahashi, Terutoshi Sakakura, Takashi Komori, Kiyoaki Tanaka

**Affiliations:** a Graduate School of Materials Science and Engineering, Nagoya Institute of Technology, Gokiso-cho, Showa-ku, Nagoya, Japan

## Abstract

The crystal structure of EuRu_4_P_12_ is isotypic with filled skutterudite structures of rare earth transition metal poly­phosphides: *R*Fe_4_P_12_ (*R* = Ce, Pr, Nd, Sm and Eu), *R*Ru_4_P_12_ (*R* = La, Ce, Pr and Nd) and *R*Os_4_P_12_ (*R* = La, Ce, Pr and Nd). The Ru cation is coordinated by six P anions in a distorted octa­hedral manner. The partially occupied Eu position (site occupancy 0.97) is enclosed by a cage formed by the corner-shared framework of the eight RuP_6_ octa­hedra.

## Related literature

The title compound is isotypic with the *Im* 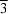 form of LaFe_4_P_12_, see: Jeitschko & Braun (1977 ▶). For the single-crystal preparation and magnetic and electrical properties of EuRu_4_P_12_, see: Sekine *et al.* (2000 ▶). For hyperfine inter­action in EuRu_4_P_12_, see: Grandjean *et al.* (1983 ▶); Indoh *et al.* (2002 ▶). For the method used to avoid multiple diffraction, see: Takenaka *et al.* (2008 ▶).

## Experimental

### 

#### Crystal data

Eu_0.97_Ru_4_P_12_
*M* *_r_* = 923.37
Cubic, 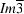

*a* = 8.04163 (10) Å
*V* = 520.04 (1) Å^3^
*Z* = 2
Mo *K*α radiation
μ = 13.37 mm^−1^
*T* = 100 K
0.04 mm (radius)

#### Data collection

MacScience M06XHF22 four-circle diffractometer
Absorption correction: for a sphere [transmission coefficients for spheres tabulated in International Tables Vol. C (1992), Table 6.3.3.3, were interpolated with Lagrange’s method (four-point interpolation; Yamauchi *et al.*, 1965 ▶)] *T*_min_ = 0.486, *T*_max_ = 0.526
1564 measured reflections
769 independent reflections
625 reflections with *F* > 3σ(*F*)
*R*_int_ = 0.016

#### Refinement

*R*[*F* ^2^ > 2σ(*F* ^2^)] = 0.020
*wR*(*F* ^2^) = 0.024
*S* = 1.54
1304 reflections
30 parameters
Δρ_max_ = 2.08 e Å^−3^
Δρ_min_ = −1.14 e Å^−3^

### 

Data collection: *MXCSYS* (MacScience, 1995 ▶) and *IUANGLE* (Tanaka *et al.*, 1994 ▶); cell refinement: *RSLC-3* *UNICS* system (Sakurai & Kobayashi, 1979 ▶); data reduction: *RDEDIT* (Tanaka, 2008 ▶); program(s) used to solve structure: *QNTAO* (Tanaka *et al.*, 2008 ▶); program(s) used to refine structure: *QNTAO*; molecular graphics: *ATOMS for Windows* (Dowty, 2000 ▶); software used to prepare material for publication: *RDEDIT*.

## Supplementary Material

Crystal structure: contains datablocks global, I. DOI: 10.1107/S1600536810000589/br2130sup1.cif

Structure factors: contains datablocks I. DOI: 10.1107/S1600536810000589/br2130Isup2.hkl

Additional supplementary materials:  crystallographic information; 3D view; checkCIF report

## Figures and Tables

**Table 1:** Selected bond lengths (Å)

Eu1—P1	3.1112 (3)
Eu1—Ru1	3.4821 (1)
Ru1—P1	2.3558 (1)
P1—P1^i^	2.3061 (1)
P1—P1^ii^	3.0829 (1)

Symmetry codes: (i) 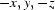; (ii) 
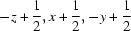
.

**Fig. 1.**
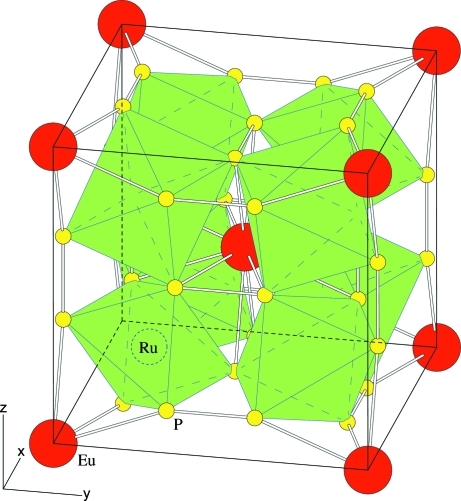
The structure of EuRu4P12 at 100 K. Small yellow and large red spheres, respectively, represent P and Eu atoms. Green distorted octahedron represent RuO6 units.

**Fig. 2.**
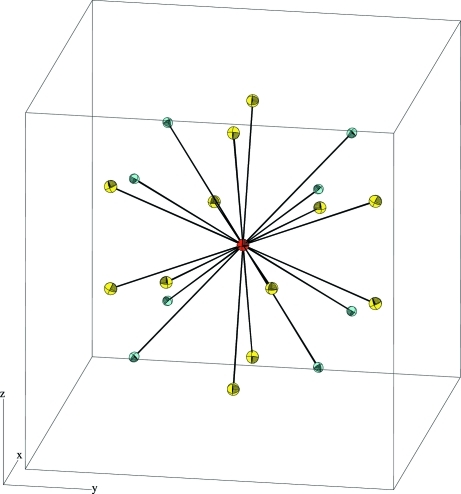
Bonding of Ru4 and P12 around an Eu ion with displacement ellipsoids at the 90% probability level. Red, blue and yellow ellipsoids represent Eu, Ru and P atoms, in Fig. 1.

## supplementary crystallographic information

### Refinement

Multiple diffraction was avoided by using ψ-scans (Takenaka *et al.*, 2008). Intensities were measured at the equi-temperature region of combination of angles ω and χ of a four-circle diffractometer. The intensities have not been included for the refinement if the multiple diffraction cannot be avoided. In addition, the crystal was cooled to 100 K with an Oxford cryostream cooler installed on a four-circle diffractometer. Since the temperature of the sample depends on the ω and χ-angle and the X-ray diffraction measurement was carried out in the equi-temperature area, the ω and χ-angle had the limitation. Thus completeness of the independent reflection was less than 85%.

### Crystal data

**Table:**

Eu_0.97_Ru_4_P_12_	*D*_x_ = 5.925 Mg m^−^^3^
*M**_r_* = 923.37	Mo *K*α radiation, λ = 0.71073 Å
Cubic, *I**m*3	Cell parameters from 37 reflections
Hall symbol: -I 2 2 3	θ = 36.0–37.7°
*a* = 8.04163 (10) Å	µ = 13.37 mm^−^^1^
*V* = 520.04 (1) Å^3^	*T* = 100 K
*Z* = 2	Sphere, black
*F*(000) = 828.4	0.04 mm (radius)

### Data collection

**Table:**

MacScience M06XHF22 four-circle diffractometer	769 independent reflections
Radiation source: fine-focus rotating anode	625 reflections with *F* > 3σ(*F*)
graphite	*R*_int_ = 0.016
Detector resolution: 1.25 x 1.25° pixels mm^-1^	θ_max_ = 74.2°, θ_min_ = 3.6°
ω/2θ scans	*h* = −18→20
Absorption correction: for a sphere [transmission coefficients for spheres tabulated in International Tables Vol. C (1992), Table 6.3.3.3, were interpolated with Lagrange's method (four-point interpolation; Yamauchi *et al.*, 1965)]	*k* = −21→21
*T*_min_ = 0.486, *T*_max_ = 0.526	*l* = −18→20
1564 measured reflections	

### Refinement

**Table:**

Refinement on *F*	Weighting scheme based on measured s.u.'s
Least-squares matrix: full	(Δ/σ)_max_ = 0.018
*R*[*F*^2^ > 2σ(*F*^2^)] = 0.020	Δρ_max_ = 2.08 e Å^−^^3^
*wR*(*F*^2^) = 0.024	Δρ_min_ = −1.14 e Å^−^^3^
*S* = 1.54	Extinction correction: B–C type 1 Gaussian isotropic (Becker & Coppens, 1975)
1304 reflections	Extinction coefficient: 0.068 (6)
30 parameters	

### Fractional atomic coordinates and isotropic or equivalent isotropic displacement parameters (Å^2^)

**Table:**

	*x*	*y*	*z*	*U*_iso_*/*U*_eq_	Occ. (<1)
Eu1	0.000000	0.000000	0.000000	0.00270 (2)	0.970 (4)
Ru1	0.250000	0.250000	0.250000	0.001840 (15)	
P1	0.000000	0.359329	0.143386	0.00283 (4)	

### Atomic displacement parameters (Å^2^)

**Table:**

	*U*^11^	*U*^22^	*U*^33^	*U*^12^	*U*^13^	*U*^23^
Eu1	0.00271 (3)	0.00271 (3)	0.00271 (3)	0	0	0
Ru1	0.00185 (3)	0.00185 (3)	0.00185 (3)	0.000108 (17)	0.000108 (17)	0.000108 (17)
P1	0.00268 (10)	0.00301 (10)	0.00285 (10)	0	0	−0.00009 (7)

### Geometric parameters (Å, °)

**Table:**

Eu1—P1	3.1112 (3)	Ru1—P1^i^	2.3558 (1)
Eu1—Ru1	3.4821 (1)	P1—P1^ii^	2.3061 (1)
Ru1—P1	2.3558 (1)	P1—P1^i^	3.0829 (1)
			
Eu1—P1—Ru1	77.78	Ru1—P1—P1^i^	49.13
Eu1—P1—P1^ii^	68.25	P1—Ru1—P1^i^	81.74
Eu1—P1—P1^i^	109.77	P1^ii^—P1—P1^i^	89.59
Ru1—P1—P1^ii^	111.34		

Symmetry codes:  (i) −*z*+1/2, *x*+1/2, −*y*+1/2; (ii) −*x*, *y*, −*z*.
